# Exploring shotgun metagenomic data to detect microeukaryotic pathogens in wildlife

**DOI:** 10.1186/s12866-026-05298-9

**Published:** 2026-06-25

**Authors:** Holly Nichols, Aleksey Molokin, Cary Pirone Davies, Jenny G. Maloney

**Affiliations:** 1 Department of Agriculture, Environmental Microbial and Food Safety Laboratory, Agricultural Research Service, Beltsville, MD 20705 USA; 2 Department of Bacteriology, University of Wisconsin-Madison, Madison, WI 53706 USA; 3 Microbiology Doctoral Training Program, University of Wisconsin-Madison, Madison, WI 53706 USA; 4 Department of Agriculture, Animal Biosciences and Biotechnology Laboratory, Agricultural Research Service, Beltsville, MD 20705 USA

**Keywords:** Shotgun metagenomics, Microeukaryotes, Microeukaryotic parasites, Wildlife, Kraken 2, Goose

## Abstract

**Background:**

Microeukaryotic parasites of the intestinal tract are an understudied group of organisms that infect humans and many other animals. Targeted sequencing methods focused on individual loci are usually employed for detection of these parasites, making comprehensive studies of microeukaryotic parasite diversity within hosts or other systems difficult. Exploratory approaches such as shotgun metagenomic sequencing to survey the diversity of microeukaryotic parasites in new and existing datasets are not well developed.

**Results:**

Utilizing existing datasets from 12 goose fecal samples, we explored some of the benefits and challenges of using shotgun metagenome sequencing to detect microeukaryotic parasites. We demonstrated the importance of careful curation of read classification data to avoid erroneously linking pathogens to hosts or environments as unsupported classifications were common in the data and varied widely depending on analysis parameters. However, we were able to establish strong support for the presence of sequences of *Eimeria* and *Enterocytozoon bieneusi*. In addition, examination of trichomonad reads indicated that parasite reads mapping to human pathogens unlikely to colonize geese may in fact represent cryptic microeukaryotic species that are not included in existing curated databases opening new potential avenues of study.

**Conclusions:**

Taken together these findings support the idea that exploring microeukaryotic parasite diversity within shotgun metagenomic datasets can be beneficial to our understanding of the presence and diversity of these organisms in wildlife hosts.

**Supplementary Information:**

The online version contains supplementary material available at https://doi.org/10.1186/s12866-026-05298-9.

## Background

Intestinal microeukaryotic parasites are a diverse group of organisms that are responsible for diseases that impact humans and many other animals [1]. The presence and transmission of microeukaryotic parasites are of concern to public and animal health and can negatively impact wildlife populations and animal agriculture. Detection of microeukaryotic parasites in fecal and environmental samples is an important component of successful intervention strategy development and disease control. However, detection of these parasites can be an inherently difficult process. Factors that complicate detection include low abundance of parasite forms in samples, life stages that are difficult to differentiate from other artifacts through microscopic observation, and the lack of culture-based methods for propagation of many species. Thus, molecular detection methods that leverage the sensitivity and specificity of PCR have become the gold standard in accurate and rapid parasite detection [2]. By pairing PCR based detection with Sanger sequencing for sequence-based parasite identification, many advances in our understanding of host specificity, disease severity, and disease ecology have been achieved. The ability to survey DNA in diverse host groups as well as the environment has greatly expanded our understanding of the diversity of microeukaryotes [3, 4].

In the current era of DNA sequencing, the increasing affordability of massively parallel, high throughput next generation sequencing (NGS) has allowed for the emergence of the field of metagenomics, which allows for the direct sequencing of all DNA obtained from an environment [5]. Metagenomic sequencing can be based on the amplification of specific loci already in use for the detection and differentiation of a taxonomic group of interest, allowing for the detection of co-infecting lineages of parasites within an individual sample [6–9]. This type of amplicon based NGS has already shown promise for expanding our understanding of the diversity of known parasite populations within individual samples and hosts [10–20]. However, the diversity of microeukaryotic parasites makes detection of undescribed parasites through amplicon sequencing difficult [21]. Even when employing the small subunit rRNA (SSU rRNA) gene 18S in eukaryotes, which is frequently used to profile microbial eukaryotes, multiple primer sets are often required to capture the full phylogenetic diversity of this group [1]. Untargeted approaches can be used to explore total microeukaryotic diversity without a priori assumptions required by primer choice. Shotgun metagenomic sequencing is achieved through NGS of all DNA present within a sample. This approach does not rely on primer availability, the need for culture, or any other organism specific enrichment step and can be used to survey the entire variety of DNA-based life forms in a sample. Additionally, unlike amplicon-based methods, shotgun sequencing is not prone to amplification biases that may skew read abundances. As such, shotgun sequencing data hold promise for revealing the presence and diversity of microeukaryotes, especially those microeukaryotic pathogens in host populations that may be difficult to study.

Wildlife populations are colonized by a diversity of understudied microeukaryotes. These microeukaryotes may negatively impact host populations, but even relatively mild infections should be monitored as wild animals are reservoir hosts for parasite species with zoonotic potential or with potential to impact agriculture. Despite this potential, there is little work that has been done to explore the utility of shotgun sequencing for the detection and differentiation of microeukaryotic parasites in wildlife populations.

The characterization of microeukaryotes in wildlife populations and the environment is particularly relevant in a changing climate as parasites can serve as sentinels of both environmental and animal health [4, 22]. Studying parasite populations in animals can also be beneficial to public health as animals can serve as surveillance sentinels for zoonotic parasites [23]. Sampling migratory birds offers a unique opportunity as they interface between wildlife and domestic environments and can assist in the surveillance of zoonotic disease and environmental health [24–27]. Additionally, many wild birds experience high stress environments making them particularly vulnerable to negative impacts from host specific or zoonotic parasite infections [28]. The role of birds as a dispersal mechanism for microeukaryotes has previously been investigated [29]. Thus, robust characterization of parasite populations in birds represents an opportunity to achieve multiple benefits.

Exploring parasite populations in wild birds is challenging as wild birds are hard to sample. Birds may belong to protected populations, making access to fecal samples for sequence-based detection methods difficult. Therefore, we suggest that existing fecal metagenome samples should be investigated for the presence of microeukaryotic parasites to maximize utility of samples which are difficult to acquire. To that end, we explore the use of shotgun metagenomic sequence data for the identification of microeukaryotic intestinal pathogens in birds by utilizing two previously published data sets from goose fecal samples from the U.S. and China. Some of the challenges associated with accurate microeukaryote identification in this type of data are explored and discussed.

## Methods

### Data sources

Samples were selected based on a Google Scholar literature search using combinations of keywords ‘avian’, ‘feces’, ‘migratory’, and ‘shotgun metagenome’. A total of 11 publications representing 228 shotgun metagenome samples were considered. Projects were discarded if sample collection or DNA extraction enriched for bacterial cells (differential centrifugation, passage through filter, etc.) or if avian samples were non-migratory. Shotgun metagenomic datasets from fecal samples of eight Canada geese (*Branta canadensis*) from the Northeastern United States generated by Gil and Hird, 2022 (BioProject ID PRJNA879251; Run IDs SRR21527943, SRR21527944, SRR21527945, SRR21527946, SRR21527947, SRR21527948, SRR21527967, SRR21527968) and from four Bar-headed geese (*Anser indicus*) from Qinghai, China generated by Wang et al., 2017 (BioProject IDs PRJNA316560 and PRJNA316570; Run IDs SRR3327343, SRR3327411, SRR3327572, SRR3327573) were retrieved from the National Center for Biotechnology Information, Sequence Read Archive [30, 31]. Total raw reads for each goose fecal sample included in the present study are listed with corresponding run numbers in Table 1. Institutional Review Board approval was not required for this study as only publicly available data were utilized for analyses.

**Table 1:** Effect of confidence threshold on the percentage of reads classified by Kraken 2 within each sample

		Confidence thresholds tested			
Sample run ID	No. raw reads	0.05	0.10	0.50	0.75
SRR3327343	18,033,417	0.06	0.008	0.0004	0.0002
SRR3327411	22,333,374	0.06	0.02	0.0005	0.0003
SRR3327572	22,366,453	0.05	0.006	0.0007	0.0006
SRR3327573	20,980,311	0.06	0.009	0.002	0.002
SRR21527943	19,417,800	0.1	0.02	0.001	0.0005
SRR21527944	20,563,704	0.1	0.07	0.02	0.009
SRR21527945	15,413,737	0.3	0.2	0.04	0.007
SRR21527946	46,304,011	0.4	0.2	0.02	0.01
SRR21527947	17,137,210	0.08	0.03	0.003	0.002
SRR21527948	15,483,508	0.06	0.02	0.002	0.0008
SRR21527967	21,498,732	0.3	0.2	0.02	0.009
SRR21527968	15,422,368	0.1	0.07	0.01	0.002

### Taxonomic classification of reads

Quality control of raw sequencing data was performed using Trimmomatic software and included the removal of reads shorter than 36 bps and trimming low quality or N bases (below quality 3) [32]. Read classification was performed using Kraken 2 v2.1.3 and the pre-built Kraken 2 database EuPathDB46-Clean [33, 34]. Except for varying confidence threshold, default parameters were used. The EuPathDB46-Clean database was selected because it contains a curated set of eukaryotic pathogen genomes, including microeukaryotes that are important pathogens of both humans and wildlife and has been processed to remove contaminating and low-complexity sequences (database available at https://ccb.jhu.edu/data/eupathDB/) [35]. The influence of confidence threshold selection on read classification was assessed by evaluating confidence thresholds of 0.05, 0.1, 0.5, and 0.75 (Additional file 1). Because the confidence threshold of 0.5 performed best for minimizing read matches to organisms unlikely to be observed in fecal specimens while limiting the potential decrease in sensitivity from using higher confidence thresholds, reads classified at this threshold were selected for further analyses by BLAST using the core_nt database [36]. Classified reads from each sample were extracted and further examined by BLAST to support taxonomic classification and identification of microeukaryotic pathogen sequences (Additional file 2). The BLAST core nucleotide database was used to assess all Kraken 2 classified reads. From the BLAST results for each sample, only reads identified as Eukaryota at the superkingdom level with ≥ 98% sequence identity between read and BLAST hit were retained to assess agreement between BLAST and Kraken 2 species level assignments. Coverage was not used as an exclusion criterion as most BLAST hits were at or near full-length. The full BLAST table for each sample included in the study can be found in additional file 2.

## Results

Shotgun metagenomic datasets from fecal samples collected from 12 geese were analyzed for the presence of microeukaryotic pathogens using Kraken 2 and the EuPathDB46-Clean database. Kraken 2 uses a k-mer based approach for read classification. Reads are broken into short sequences (k-mers), each is mapped to a reference database, and given a taxon ID; the taxon with the highest proportion of k-mer matches is assigned to the read; users can select a confidence threshold that can range from 0 to 1 [37]. For example, when a user identified threshold of 0.1 is used, the fraction of the majority taxon must be more than 10% for the read to be accepted. Kraken 2 uses a default confidence threshold of 0 for read classification. However, this threshold may not be ideal for microeukaryotic pathogen detection as even a single k-mer hit is enough to erroneously classify a read. To determine how threshold selection influenced read classification in this dataset, we assayed four confidence thresholds (0.05, 0.1, 0.5, and 0.75) and compared read classifications from each threshold.

**Fig. 1:**
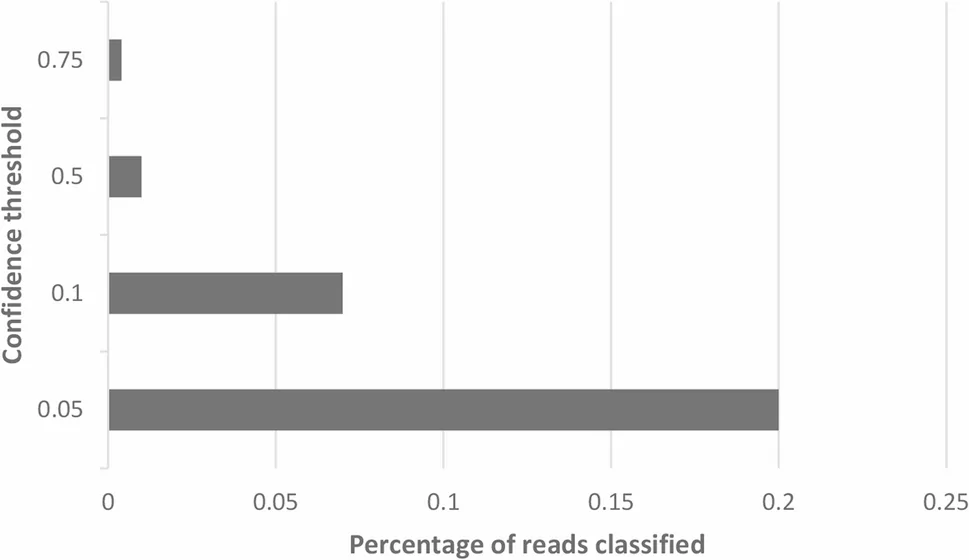
Average percentage of reads classified at each tested confidence threshold among the 12 samples from geese

Increasing confidence threshold decreased the percentage of reads classified by Kraken 2 in each sample assayed (Table 1). Of the 254,954,625 raw reads in the initial dataset included in this study, 0.2%, 0.07%, 0.01%, and 0.004% reads were classified at confidence thresholds of 0.05, 0.1, 0.5, and 0.75, respectively (Table 1 and Fig. 1). As a first pass assessment of the taxonomic classifications within each of these confidence thresholds, we chose to compare reads classified to five genera. *Cyclospora* was selected because while it is a fecally transmitted pathogen, the species *Cyclospora cayetanensis*, which was frequently observed in the classified reads, is considered host specific to humans. *Eimeria* was selected as it is fecally transmitted and a common parasite of avian species. *Leishmania*, *Plasmodium*, and *Trypanasoma* were assessed as these are genera of blood and tissue borne pathogens whose presence in goose feces should be minimal. At a confidence threshold of 0.05, there were 215 reads classified to *Cyclospora cayetanensis* among the 12 samples (Table 2). Only one read was classified as *Cyclospora cayetanensis* at confidence thresholds of 0.5 and 0.75. Similarly, *Leishmania* and *Plasmodium* were reduced from 382 to 912 reads at 0.05 confidence threshold to 0 and 3 reads at a confidence threshold of 0.5. *Trypanosoma* which had 20,567 reads at 0.05 was reduced to only 1 read at a confidence threshold of 0.5. Reads classified to the parasite *Eimeria*, which has several avian specific species, were also greatly reduced by increasing the confidence threshold used for taxonomic classification. There were 43,120 reads classified as *Eimeria* at a confidence threshold of 0.05. However, there were still 7,739 and 5,212 reads classified as Eimeria at 0.5 and 0.75 confidence thresholds, respectively (Table 2). To balance sensitivity and specificity among the five genera a confidence threshold of 0.5 (> 50% of k-mers assigned to the majority taxon) was chosen to minimize spurious classifications before further analysis by BLAST to bolster support for microeukaryotic pathogens detected within the samples.

**Table 2:** Number of reads assigned to five select genera at tested confidence thresholds among the 12 samples from geese

	Confidence thresholds tested			
	0.05	0.1	0.5	0.75
*Cyclospora*	215	59	1	1
*Eimeria*	43,120	28,520	7739	5212
*Leishmania*	382	32	0	0
*Plasmodium*	912	98	3	0
*Trypanasoma*	20,567	262	1	0

**Fig. 2:**
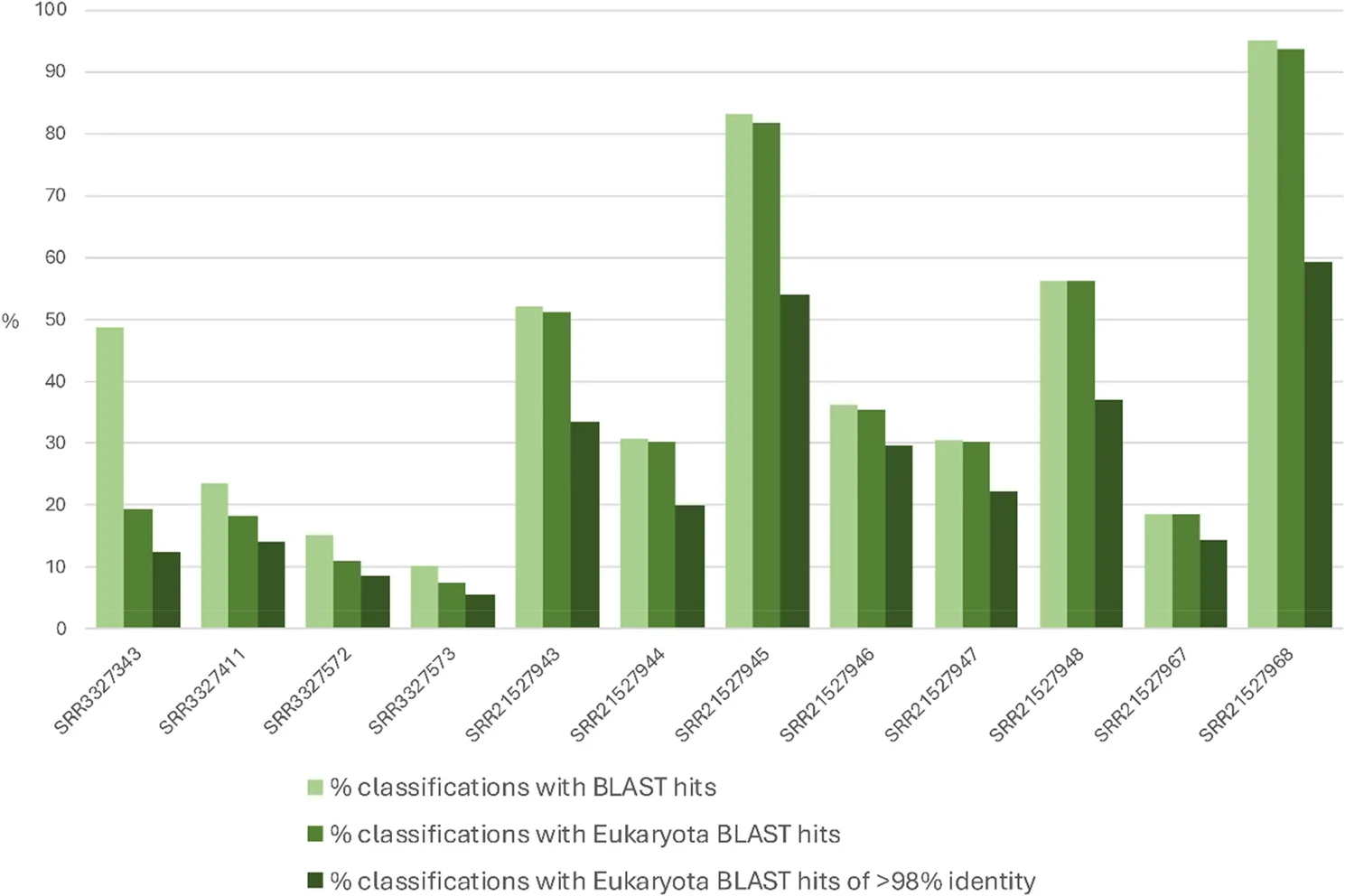
Comparing Kraken 2 0.5 confidence threshold read classifications with BLAST in each goose sample

All reads classified by Kraken 2 at the 0.5 confidence threshold across the 12 geese fecal samples were extracted and analyzed by BLAST. The percentage of Kraken 2 classified reads that also returned BLAST hits varied widely by sample from 10.2 to 95.1% (Fig. 2). Parsing the BLAST hits showed a slightly lower percentage of Kraken 2 classified reads returning a hit to Eukaryota. When Eukaryotic BLAST hits were filtered for > 98% sequence identity the percentage of Kraken 2 classified reads fell sharply and ranged from 5.4% to 59.3% among the samples.

There were seven microeukaryotic pathogens with fecal transmission pathways observed in the classifications at 0.5 confidence threshold (Table 3). We chose to focus on these organisms because their presence in fecal samples is relevant to the health of the animals as well as to the potential for the animals to serve as reservoirs for these organisms. Of the seven microeukaryotic pathogens identified in the Kraken 2 analysis, only three were supported by reads classified to the same species or genera by the BLAST analysis (Table 3). The organisms with shared support between the two read classification methods included two avian parasites, *Cryptosporidium baileyi* and *Eimeria*, and one potentially zoonotic pathogen *Enterocytozoon bieneusi*. However, it is important to note that *C. baileyi* was only supported by a single read in the BLAST analysis.

**Table 3:** Microeukaryotic pathogens with fecal transmission pathways classified by Kraken 2 and supported by BLAST in each sample

Sample run ID	Eukaryotic intestinal pathogens identified at 0.5 confidence threshold (No. of reads)	Supporting reads in > 98% Eukaryota BLAST (No. of reads)
SRR3327343	*Eimeria* (3)	
	*Giardia lamblia* (9)	
SRR3327411	*Eimeria* (54)	
	*Giardia lamblia* (1)	
	*Enterocytozoon bieneusi* (2)	
SRR3327572	*Eimeria* (124)	
SRR3327573	*Eimeria* (337)	
SRR21527943	*Eimeria* (42)	
	*Enterocytozoon bieneusi* (1)	
SRR21527944	*Eimeria* (93)	*Eimeria* (5)
	*Enterocytozoon bieneusi* (3092)	*Enterocytozoon bieneusi* (44)
SRR21527945	*Cryptosporidium baileyi* (1042)	*Cryptosporidium baileyi* (1)
	*Eimeria* (23)	
	*Enterocytozoon bieneusi* (5)	*Enterocytozoon bieneusi* (2)
SRR21527946	*Eimeria* (4011)	*Eimeria* (19)
	*Cyclospora cayetanensis* (1)	
	*Enterocytozoon bieneusi* (1)	
SRR21527947	*Eimeria* (355)	*Eimeria* (2)
	*Entamoeba histolytica* (1)	
SRR21527948	*Eimeria* (117)	*Eimeria* (1)
SRR21527967	*Eimeria* (2579)	*Eimeria* (72)
	*Hammondia hammondi* (1)	
SRR21527968	*Eimeria* (1)	*Eimeria* (1)
	*Enterocytozoon bieneusi* (66)	*Enterocytozoon bieneusi* (1)

Another human pathogen that was frequently observed in the Kraken 2 dataset with high read support was *Trichomonas vaginalis*. Even at a confidence threshold of 0.5, *T. vaginalis* was supported by over 1,000 reads in two samples (Table 4). While *T. vaginalis* is an unlikely finding in goose fecal samples, other trichomonad species are commonly observed in Anseriformes, and indeed, several trichomonad species were identified by the BLAST analysis of Kraken 2 classified reads. Notably, *Tetratrichomonas gallinarum*, a trichomonad commonly found in Anseriformes was one of the most common classifications in the BLAST results (Table 4). However, *T. vaginalis* was still supported by a large number of reads by BLAST with seven of the nine samples that contained trichomonad reads having more reads classified as *T. vaginalis* by BLAST than any other trichomonad species. We also observed the important bird pathogen *Histomonas meleagridis* among the results. It is important to specify that there were a number of reads that matched this taxon in the BLAST results and that the reads classified to this species represent conserved gene regions that cannot be differentiated from other trichomonad species. Thus, the observation of *H. meleagridis* is unlikely to be a legitimate finding and further supports the need for careful curation of metagenomic data.

**Table 4:** Trichomonads classified by Kraken 2 and supported by BLAST in each sample

Sample run ID	Trichomonads identified by Kraken 2 at 0.5 confidence	Trichomonads identified in > 98% Eukaryota by BLAST
SRR3327411	*Trichomonas vaginalis* (4 reads)	*Tetratrichomonas gallinarum* (2 reads)
		*Trichomonas vaginalis* (1 read)
SRR21527943	*Trichomonas vaginalis* (75 reads)	*Trichomonas vaginalis* (43 reads)
		*Tetratrichomonas gallinarum* (6 reads)
		*Tetratrichomonas prowazeki* (2 reads)
		*Tetratrichomonas* sp. (2 reads)
SRR21527944	*Trichomonas vaginalis* (722 reads)	*Trichomonas vaginalis* (389 reads)
		*Tetratrichomonas gallinarum* (345 reads)
		*Tetratrichomonas* sp. (31 reads)
		*Tetratrichomonas buttreyi* (16 reads)
		*Trichomonas* sp. (4 reads)
		*Tetratrichomonas prowazeki* (2 reads)
		*Tetratrichomonas undula* (1 read)
		*Simplicimonas similis* (3 reads)
		*Simplicimonas* sp. (1 read)
SRR21527945	*Trichomonas vaginalis* (2876 reads)	*Tetratrichomonas gallinarum* (1673 reads)
		*Trichomonas vaginalis* (1450 reads)
		*Tetratrichomonas* sp. (145 reads)
		*Tetratrichomonas buttreyi* (98 reads)
		*Simplicimonas similis* (29 reads)
		*Trichomonas* sp. (24 reads)
		*Tetratrichomonas prowazeki* (10 reads)
		*Tetratrichomonas limacis* (4 reads)
		*Histomonas meleagridis* (2 reads)
		*Monocercomonas* sp. (2 reads)
		*Simplicimonas* sp. (2 reads)
		*Pentatrichomonas hominis* (1 read)
		*Pseudotrichomonas* sp. (1 read)
SRR21527946	*Trichomonas vaginalis* (272 reads)	*Trichomonas vaginalis* (132 reads)
		*Tetratrichomonas gallinarum* (76 reads)
		*Tetratrichomonas* sp. (16 reads)
		*Tetratrichomonas prowazeki* (4 reads)
		*Simplicimonas similis* (3 reads)
		*Tetratrichomonas buttreyi* (2 reads)
		*Tetratrichomonas undula* (2 reads)
		*Pentatrichomonas hominis* (1 read)
		*Tetratrichomonas limacis* (1 read)
SRR21527947	*Trichomonas vaginalis* (90 reads)	*Trichomonas vaginalis* (56 reads)
		*Tetratrichomonas gallinarum* (21 reads)
		*Tetratrichomonas prowazeki* (8 reads)
		*Tetratrichomonas buttreyi* (1 read)
		*Tetratrichomonas* sp. (7 reads)
SRR21527948	*Trichomonas vaginalis* (97 reads)	*Trichomonas vaginalis* (44 reads)
		*Tetratrichomonas gallinarum* (29 reads)
		*Tetratrichomonas* sp. (7 reads)
		*Tetratrichomonas prowazeki* (2 reads)
		*Pseudotrichomonas* sp. (1 read)
		*Trichomonas* sp. (1 read)
SRR21527967	*Trichomonas vaginalis* (114 reads)	*Trichomonas vaginalis* (56 reads)
		*Tetratrichomonas gallinarum* (19 reads)
		*Tetratrichomonas* sp. (6 reads)
		*Tetratrichomonas prowazeki* (4 reads)
		*Tetratrichomonas buttreyi* (2 reads)
		*Pentatrichomonas hominis* (1 read)
SRR21527968	*Trichomonas vaginalis* (1021 reads)	*Trichomonas vaginalis* (517 reads)
		*Tetratrichomonas gallinarum* (488 reads)
		*Tetratrichomonas* sp. (52 reads)
		*Tetratrichomonas buttreyi* (41 reads)
		*Tetratrichomonas limacis* (8 reads)
		*Simplicimonas similis* (7 reads)
		*Trichomonas* sp. (6 reads)
		*Tetratrichomonas prowazeki* (5 reads)
		*Tetratrichomonas undula* (2 reads)
		*Histomonas meleagridis* (1 read)

## Discussion

Metagenomic data is an increasingly available resource with many potential uses including surveying the presence of microeukaryotic pathogens in host and environmental samples. However, the processing of this data can be a challenge as taxonomic classification of the millions of reads produced in a shotgun sequencing run can be computationally intensive. BLAST is considered the gold standard for sequence classification but is often not feasible for large scale metagenomics studies because of time and computational needs required for the assessment of millions of reads [38]. Tools for metagenomic analysis that use k-mer based classification systems provide an alternative that makes assessment of sample composition more feasible [38]. Kraken 2 has emerged as a popular tool for the classification of metagenomic sequence data in part because the k-mer based read classification method it employs can be performed relatively quickly while consuming less computational resources [33, 34]. Another issue with classification of microeukaryotic pathogens from shotgun metagenomic datasets is the lack of curated reference sequences available for these organisms to populate databases used in sequence classification. Thus, database choice can be an important factor influencing the reliability of classification results [37, 39]. Here we used Kraken 2 and the EuPathDB46-Clean database to assess the utility of these tools for classification of microeukaryotic pathogens in shotgun metagenome data from 12 goose fecal samples.

We first assessed the effect of Kraken 2 confidence threshold selection on read classification. As confidence threshold increases, a larger portion of each read must match a given reference to result in classification to that taxonomic level. Increasing the confidence threshold can decrease misclassifications but can also potentially decrease sensitivity [37]. Thus, the choice of confidence threshold for a study can be useful in improving the accuracy of the analysis of shotgun sequence data. We tested four confidence thresholds, 0.05, 0.1, 0.5, and 0.75, and compared the percentage of reads with taxonomic assignments at each level (Table 1 and Fig. 1). We also selected five microeukaryotic pathogen genera to track across the four confidence threshold levels as a measure of performance of each threshold (Table 2). We found that confidence threshold selection is an important consideration in shotgun sequencing studies using Kraken 2 for microeukaryotic pathogen identification. There was a striking decrease in the number of reads classified with increasing confidence level (Table 2). At lower confidence thresholds of 0.05 and 0.1, reads classified to non-fecal pathogens *Leishmania*, *Plasmodium*, and *Trypanosoma*, and to the host specific pathogen *Cyclospora cayetanensis* were observed at levels that could support the identification of these parasites in the data. However, by increasing the confidence threshold to 0.5 and 0.75, reads classified to these organisms were reduced to 3 or fewer (Table 2). While more stringent confidence thresholds did appear to positively impact the accuracy of read classification using Kraken 2, further analysis of Kraken 2 classified reads using BLAST indicated that it does not eliminate potentially erroneous classifications from the data set. In fact, even when using a confidence threshold of 0.5, only between 5.4 and 59.3% of Kraken 2 classified reads were classified as Eukaryotic by BLAST when BLAST hits were filtered for > 98% sequence identity (Fig. 2). This may occur for a variety of reasons including differences in the databases used and the way in which classifications are assigned. Overall, selection of confidence threshold is important when using Kraken 2, and Kraken 2 alone may not be suitable for microeukaryotic pathogen detection. Other supporting data, especially when reporting unexpected pathogens, should be considered.

The comparison of Kraken 2 classified reads and BLAST classifications did support the presence of three microeukaryotic pathogens in the goose fecal samples analyzed in this study. Two avian parasites, *Cryptosporidium baileyi* and *Eimeria*, and one potentially zoonotic pathogen *Enterocytozoon bieneusi* were identified by Kraken 2 and had supporting reads with > 98% sequence identity in the BLAST analysis, although *C. baileyi* was only supported by a single read (Table 3). The four other intestinal microeukaryotic pathogens classified by Kraken 2 using a 0.5 confidence threshold were not supported by BLAST. Taken together these findings indicate that Kraken 2 can be a useful first step in the identification of microeukaryotic pathogens in shotgun sequencing data, but users should support Kraken 2 classifications through further analyses or be conservative in the reporting of data based on Kraken 2 classification alone.

These findings are important to consider as studies utilizing shotgun sequence data to identify microeukaryotic pathogens become more available. Two recent studies of eukaryotic microbes in environmental and waste water samples used shotgun sequencing and Kraken 2 with default settings (i.e. a confidence threshold of 0) for read classification and identified a wide array of microeukaryotic pathogens including *Cryptosporidium*, *Cyclospora*, *Giardia*, *Trypanosoma*, and *Plasmodium* [40, 41]. These results are strikingly similar to what was observed using only low confidence threshold data in the present study, supporting the importance of drawing attention to issues related to microeukaryote read classification.

Another interesting finding in the data analyzed in this study was the high level of read support for *T. vaginalis* among several of the goose samples in the Kraken 2 data. While *T. vaginalis* was the only trichomonad species identified in the samples using Kraken 2, this finding is likely an issue related to the database used for read classification: *T. vaginalis* is the only trichomonad species present in the database [42]. *Trichomonas vaginalis* is a parasite of the human reproductive tract but is a relative of trichomonad species that parasitize geese. Thus, Kraken 2 classification to *T. vaginalis* could represent misclassification to the most relevant reference in the database. Further analysis of Kraken 2 classified reads indicated that host specific trichomonad species were in fact common in the samples from geese (Table 4). *Tetratrichomonas gallinarum* is a trichomonad commonly reported in the large intestine of *Galliformes* and Anseriformes [43]. *Tetratrichomonas gallinarum* was observed in all of the samples containing reads classified as trichomonads by BLAST with high levels of read support. However, it was often second to *T. vaginalis*, with seven of the nine samples that contained trichomonad species having more *T. vaginalis* reads than *T. gallinarum* reads. Closer examination of individual reads supported as being classified to *T. vaginalis* by BLAST do not appear to be from conserved regions that cannot be distinguished from other trichomonad species as was the case with *H. meleagridis*. This classification issue could be due to a number of factors related to database deficiencies. For example, even NCBI’s core_nt database is an incomplete set of references, and as such, the *T. vaginalis* BLAST hits could represent a conserved region of a known closely related species not present in the database. Some of these reads could also represent the presence of a cryptic species of trichomonad that has yet to be identified in geese. Such a finding would further support the benefit of pursuing shotgun metagenomic sequencing studies in new hosts and environments. This type of sequencing is agnostic to the organisms present within a sample and can yield new avenues of research and expand our understanding of the diversity of organisms that can be found within a given host or environment.

The findings of this study indicate that while database choice is an important factor to consider when analyzing shotgun metagenomic sequencing data, more data from microeukaryotic species are also needed to populate read classification databases and improve their accuracy. Issues related to database curation are not unique to shotgun data. In a recent report that used 18S metagenomics to explore the diet, gut microbiota, and parasites in caribou feces, the authors also validated the results of the database they used for read classification against BLAST. Based on the results of this comparison, they opted to remove all *Cryptosporidium* classifications because of the lack of support for these classifications via BLAST [44]. Issues related to database coverage of microeukaryotes can be attributed in part to the need for more genomes and to the need for better curation of genomes from this group of organisms. It has been reported that bacterial contamination of eukaryotic genomes is a common occurrence [45]. Because many microeukaryotes are unculturable, generation of genomes from these organisms is difficult to achieve and the generation of contaminant free genomes is also a challenge. Without culture methods to amplify DNA for genome generation, microeukaryotic genomes remain underrepresented in databases. There are only approximately 10,000 genomes available of the estimated 2 million eukaryotic species [46]. Of course, this is an issue for other domains of life as well. The prokaryotes, which have much more robust database representation than eukaryotes, only have hundreds of thousands of draft genomes available compared to the estimated 1 trillion potential prokaryotic species that may exist in the world [47–50]. Clearly more genomes are needed to expand our ability to accurately classify sequence data and the addition of genomic data to sequence classification databases is likely to improve the accuracy and utility of these databases in the future.

Exploring how shotgun metagenomic sequencing might be employed to better understand microeukaryotic parasite presence and diversity was a focus of this study, and we did find that there are some potentially useful findings from this exploration. However, shotgun metagenomics may not be appropriate for all aspects of the study of microeukaryotic parasites. Microeukaryotic parasites can be a particularly challenging group of organisms to study using metagenomic methods as they often represent rare species within fecal samples. For example, in a large survey of human metagenomes it was observed that parasite sequences represented a very small portion of total sequences, averaging 0.007% of classified reads [51]. Because parasites may be present in such low abundance, it has been observed that their presence can be missed with metagenomic approaches. In a study of parasites in samples taken from waste water treatment plants, an 18S metagenomic sequencing approach failed to detect *Cryptosporidium* in any of the 26 tested samples, while a *Cryptosporidium*-specific NGS assay detected *Cryptosporidium* in all of the samples [52]. *Giardia* has also been noted as difficult to detect with metagenomic methods, as samples microscopically diagnosed as *Giardia* positive were often only supported by one or two reads in the corresponding metagenomic data [21]. Thus, studies where the detection of a specific organism is the goal will still benefit from using approaches that are highly sensitive and specific to the organism of interest. Future studies may also benefit from optimization of methods for targeting microeukaryote DNA, as DNA extractions, sequencing protocols, and classification databases optimized for prokaryotic metagenome samples might not be optimal for detecting parasites. However, the data from the present study do indicate that exploring the presence and diversity of microeukaryotes in fecal samples with shotgun metagenomics has promise for expanding our understanding of host range and potential impacts on host health of microeukaryotic pathogens.

We observed the classification of reads to three microeukaryotic intestinal pathogens that were supported by both Kraken 2 and BLAST among the analyzed samples (Table 3). The avian parasite *C. baileyi* was observed in a single sample, and although it was only supported by a single read in the BLAST analysis, its presence in geese has been reported previously, and it is considered a generalist of birds [53–55]. *Eimeria* classifications were supported by BLAST in six samples (Table 3). Although *Eimeria* appear to be common in geese, *Eimeria* of geese are not well studied [56]. *Eimeria* can be important pathogens of birds, and the pathogenic potential of some species have been reported in geese [57, 58]. The findings of this study are further supported by a recent targeted metagenomic study of zoonotic eukaryotic parasites in urban bird populations in Spain where *Cryptosporidium* spp. were observed in storks and gulls while *Eimeria* was observed in all bird species sampled [59]. Thus, shotgun metagenomic studies could be useful in identifying potential pathogens in birds or other wildlife, but studies to follow up these findings to provide more specific data on the relevance of metagenomic findings to the health of the population (for example, paired health status of the birds sampled) would be needed. We also identified the potentially zoonotic pathogen *E. bieneusi* in three of the samples analyzed in this study. *Enterocytozoon bieneusi* is common in a variety of animals and can cause disease in humans, especially the immunocompromised [60, 61]. *Enterocytozoon bieneusi* has been previously identified in geese using traditional PCR based detection methods [55, 62]. Thus, the observation of *E. bieneusi* in this study is not novel and further supports the potential use of metagenomic methods for the detection of microeukaryotic pathogens in difficult to study populations.

## Conclusions

Taken together, the findings from this study support the idea that shotgun metagenomic data could be used to detect zoonotic and host specific microeukaryotic pathogens. This may have important implications for filling data gaps as current data do not adequately address the potential for spillover between humans and birds [63]. However, the methods used to analyze metagenomic data are an important consideration, and caution should be used in reporting and interpreting results to ensure that wildlife or other hosts are not erroneously targeted as threats in the spread of disease. While parasite-specific assays remain the gold standard for accurate identification of microeukaryotic pathogens, the use of shotgun metagenomics for expanding our understanding of parasite epidemiology shows promise. Future work to generate more reference genomes, expand databases, and validate methods for microeukaryotic pathogens will help to enhance the usefulness of these types of studies and continue to improve our understanding of the role of microeukaryotic pathogens in health and disease.

## Supplementary Information

Supplementary Material 1.

Supplementary Material 2.

## Data Availability

All data generated or analyzed during this study are included in this published article and its supplementary information files.
